# Combating CHK1 resistance in triple negative breast cancer: EGFR inhibition as potential combinational therapy

**DOI:** 10.20517/cdr.2021.128

**Published:** 2022-03-08

**Authors:** Casey D. Stefanski, Jenifer R. Prosperi

**Affiliations:** ^1^Department of Biological Sciences, University of Notre Dame, Notre Dame, IN 46617, USA.; ^2^Department of Biochemistry and Molecular Biology, Indiana University School of Medicine - South Bend, South Bend, IN 46617, USA.

**Keywords:** CHK1, TNBC, EGFR, chemoresistance

## Abstract

Triple negative breast cancer (TNBC) is marked by a lack of expression of the Estrogen Receptor, Progesterone Receptor, and human epidermal growth factor receptor 2. Therefore, targeted therapies are being investigated based on the expression profiles of tumors. Due to the potential for acquired and intrinsic resistance, there is a need for combination therapy to overcome resistance. In the article by Lee et al., the authors identify that, while prexasertib (a CHK1 inhibitor) lacks efficacy alone, combination with an EGFR inhibitor provides synergistic anti-tumor effects. Advances in targeted therapy for TNBC will benefit the clinical landscape for this disease, with this study initiating a new avenue of investigation.

In the recent article by Lee *et al.*^[1]^, the primary goal of the authors was to identify a novel targeted therapy for triple negative breast cancer (TNBC). They are focused on the interaction and cooperation between epidermal growth factor receptor (EGFR) and CHK1. Prior to this study, it was well established that CHK1 inhibition (using prexasertib) increased cell death by blocking the cell cycle checkpoints. In addition, previous studies showed that signaling through the PI3K and MAPK pathways are involved in resistance to prexasertib in sarcomas. Furthermore, prexasertib has been used in combination with PI3K inhibitors to cause changes in survival and apoptosis in TNBC^[2]^. The authors mention that while prexasertib is effective as a combination therapy, its use as monotherapy has been less successful in part due to drug resistance. It’s unclear, however, whether the resistance is related to CHK1 expression, as the CHK1 expression in Supplementary Figure 1 does not appear to correlate to the efficacy of prexasertib.

In this paper, the authors test a panel of TNBC cell lines, and show that the MDA-MB-468 cells are highly resistant to prexasertib, whereas the MX-1 cells are sensitive^[1]^. Two cell lines used in this panel, MDA-MB-468 and HCC1937, are both Rb-deficient. This deficiency could impact CHK1, as a 2018 paper demonstrated that Rb-deficient cells express higher levels of CHK1 and are more sensitive to CHK1 inhibitors^[3]^. They chose to move forward with the MDA-MB-468 (resistant) and MDA-MB-231, which had moderate prexasertib sensitivity, for the remainder of their studies. They investigated the MAPK/PI3K pathways and found EGFR elevated in the resistant MDA-MB-468 cells, while AKT was elevated in both the MDA-MB-468 and the sensitive MX-1 cells. They chose to further investigate the function of EGFR in mediating prexasertib resistance, based on the expression in resistant cells. Using the MDA-MB-231 cells, they show that treatment with EGF increased resistance to prexasertib; however, it would be interesting to know whether this treatment altered receptor level or just downstream signaling. In addition, there is no dose dependence of EGF on prexasertib response, suggesting that they have reached a threshold of EGF/EGFR stimulation. As an aside, this is likely reached in the MDA-MB-468 cells as well, given that the higher dose of EGF increased sensitivity to prexasertib.

To overcome the resistance, they used a combination treatment of prexasertib with an EGFR inhibitor, erlotinib. This combination regimen showed synergy in both the resistant MDA-MB-468 and the moderately resistant MDA-MB-231 cells. Mechanistically, the authors state that there are changes in phosphorylation of Bad; however, the blots appear to show changes in the total protein. In addition to Bad, the MDA-MB-468 cells also show a decrease in phosphorylation of AKT, which is not observed in the MDA-MB-231 cells (likely due to their low baseline levels). *In vivo* studies showed that the combination of both drugs was required to decrease tumor weight in the mice injected with MDA-MB-468 cells, as opposed to single-drug treatments alone^[1]^. While these results are contradictory to the *in vitro* results from Figure 1 (reprinted from Figure 4 of Ref.^[1]^), which show that erlotinib alone could decrease cell survival in the MDA-MB-468 cells, this could be due to cell non-autonomous interactions that would be lacking *in vitro*.

**Figure 1:**
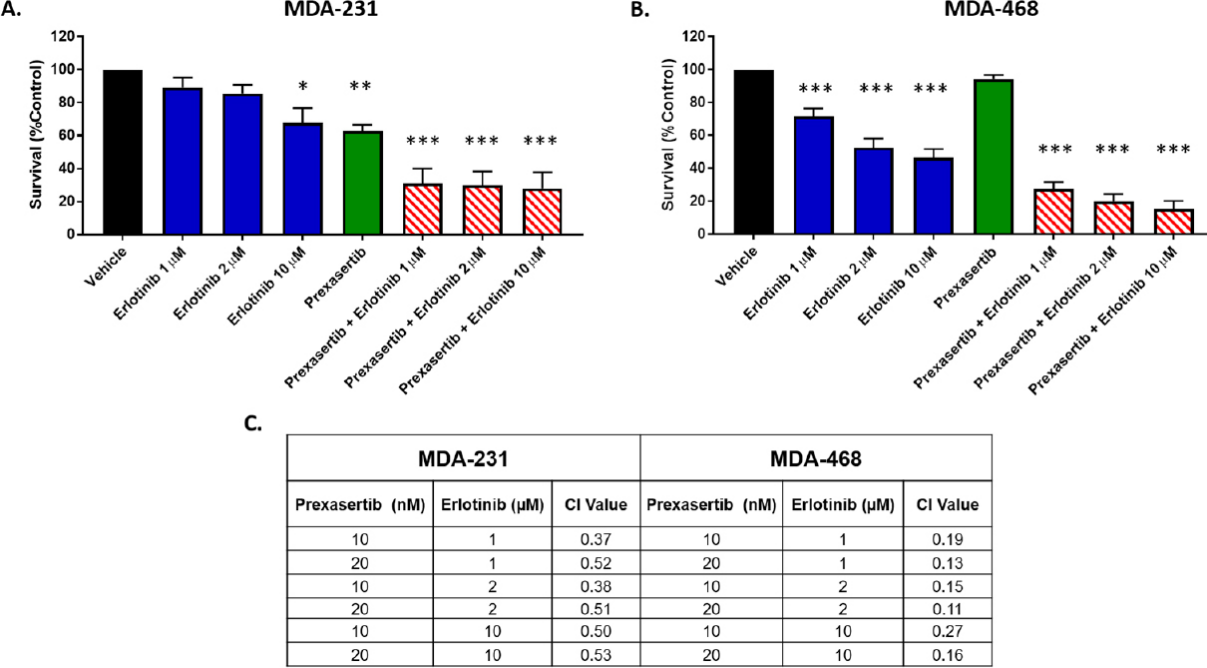
Erlotinib co-exposure with prexasertib synergistically enhanced cell killing. Viability of 3D cultures of MDA-231 (A) and MDA-468 (B) after exposure to prexasertib, erlotinib, and combinations of erlotinib and prexasertib; the combination index (CI) showed synergistic interaction (CI < 1) of erlotinib and prexasertib in both MDA-231 and MDA-468 (C).

The overall message from the manuscript is that EGFR inhibition can be used to overcome resistance to CHK1 inhibition. The manuscript by Lee *et al.*^[1]^ gives great promise and lays the foundation for further investigation into this important mechanism of treating TNBC. To aid in the understanding of the relationship between EGFR and CHK1 in TNBC, an investigation of the sensitive MX-1 cells with EGF treatment would be beneficial. A deep dive into the correlation of EGFR and CHK1 in human TNBC would also be interesting to determine potential resistance to CHK1 inhibitors. In addition, previous investigations have shown the benefit of ATR and/or PARP inhibitors with prexasertib^[4]^ (also discussed in clinical trial NCT04032080), which may provide additional investigative avenues. Regardless, the clinical need for better targeted therapies for TNBC is high, and Lee *et al.*^[1]^ have identified a novel combination regimen using EGFR inhibition with CHK1 inhibition.

## DECLARATIONS

### Copyright

© The Author(s) 2022.
