# Supplementary material for: Combating CHK1 resistance in triple negative breast cancer: EGFR inhibition as potential combinational therapy
Source: Cancer Drug Resist. 2022 Mar 8;5(1):229–32. doi: 10.20517/cdr.2021.128 (PMC8992589; doi:10.20517/cdr.2021.128)
Supplement: Supplementary file 1 [file cdr-5-229-SupplementaryMaterials.pdf]

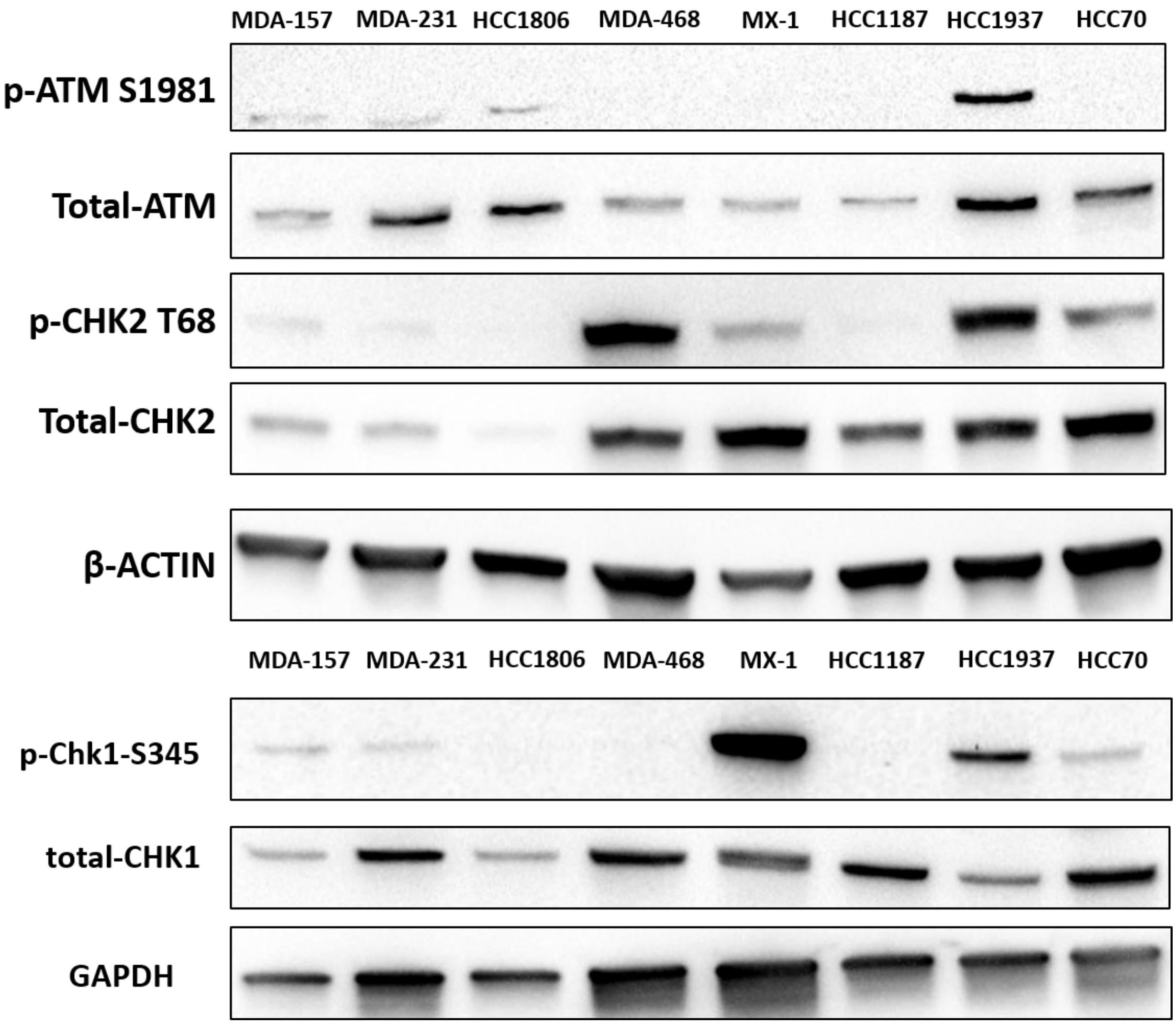

Supplementary Figure 1. Activation of DDR pathway in the TNBC cell panel. DDR: DNA damage and response; TNBC: triple negative breast cancer
